# STAPLED HEMORRHOIDOPEXY: RESULTS, LATE COMPLICATIONS, AND DEGREE OF SATISFACTION AFTER 16 YEARS OF FOLLOW-UP

**DOI:** 10.1590/0102-672020220002e1689

**Published:** 2022-09-16

**Authors:** Carlos Walter SOBRADO, Lucas Faraco SOBRADO, Carlos Almeida OBREGON, Helder Moura VILLELA, José Américo Bacchi HORA

**Affiliations:** 1 Universidade de São Paulo, Faculty of Medicine, University Hospital, Gastroenterology - São Paulo (SP), Brazil.

**Keywords:** Hemorrhoids, Hemorrhoidectomy, Surgical Staplers, Postoperative Complications, Long-Term Care, Patient Satisfaction, Hemorroidas, Hemorroidectomia, Grampeadores Cirúrgicos, Complicações Pós-Operatórias, Assistência de Longa Duração, Satisfação do Paciente

## Abstract

Stapled hemorrhoidopexy has been widely used for the treatment of hemorrhoids, but concerns about complications and recurrences after prolonged follow-up are still under debate.

**AIMS:**

The aim of this study was to evaluate the very long-term results of the stapled hemorrhoidopexy technique.

**METHODS:**

Stapled hemorrhoidopexy was performed on 155 patients between 2000 and 2003, and the early results have already been published. In this study, we evaluated the same patients after a very long follow-up. Data were collected with regard to late complications, rate and timing of recurrences, and patients’ degree of satisfaction.

**RESULTS:**

From a total of 155 patients, 98 patients were evaluated: 59 (60.2%) were interviewed by telephone and 39 (39.8%) were evaluated by outpatient consultation. The mean follow-up was 193 months (range: 184-231), 52 were female, 52 were grade III hemorrhoids, and 46 were grade IV. Recurrence was higher in grade IV (26.1%) than in grade III (7.7%) (p=0.014). Recurrence after prolonged follow-up was seen in 16 patients (16.3%) and 11 (11.2%) required reoperations. The complications were skin tags (3.1%), anal sub-stenosis (2.1%), and fecal incontinence (2.1%). After a prolonged follow-up, 82.5% of patients were either very satisfied or satisfied with the surgery.

**CONCLUSIONS:**

Stapled hemorrhoidopexy is a safe and effective treatment for hemorrhoidal disease grades III and IV. Recurrence is higher for grade IV hemorrhoids and may occur up to 9 years of follow-up. Reoperations were infrequent and there is a high patient’s degree of satisfaction associated with this technique.

## RESUMO - RACIONAL

A hemorroidopexia mecênica tem sido amplamente utilizada para o tratamento de hemorroidas, mas as preocupações com complicações e recorrências após seguimento prolongado ainda estão em debate.

**OBJETIVOS:**

Nosso objetivo foi avaliar os resultados a muito longo prazo com a técnica hemorroidopexia mecênica.

**MÉTODOS:**

O hemorroidopexia mecênica foi realizada em 155 pacientes entre 2000 e 2003, e os primeiros resultados já foram publicados. No presente estudo, avaliamos os mesmos pacientes após um seguimento muito longo. Os dados foram coletados em relação às complicações tardias, taxa e tempo de recorrência e grau de satisfação do paciente.

**RESULTADOS:**

De um total de 155 pacientes, 98 pacientes foram avaliados: 59 (60,2%) foram entrevistados por telefone e 39 (39,8%) foram avaliados por consulta ambulatorial. O seguimento médio foi de 193 meses (variação: 184-231), 52 eram do sexo feminino, 52 eram hemorroidas grau III e 46 eram grau IV. A recorrência foi maior no grau IV (26,1%) do que no grau III (7,7%) (p=0,014). A recorrência após seguimento prolongado foi observada em 16 pacientes (16,3%) e 11 (11,2%) necessitaram de reoperações. As complicações foram: plicomas (3,1%), subestenose anal (2,1%) e incontinência fecal (2,1%). Após seguimento prolongado, 82,5% dos pacientes ficaram muito satisfeitos ou satisfeitos com a cirurgia.

**CONCLUSÕES:**

O hemorroidopexia mecênica é um tratamento seguro e eficaz para a doença hemorroidária graus III e IV. A recorrência é maior para hemorroidas grau IV e pode ocorrer até 9 anos de seguimento. As reoperações foram infrequentes e há um alto grau de satisfação do paciente associado a esta técnica.

## INTRODUCTION

For many years, surgical treatment of hemorrhoidal disease (HD) consisted solely of excisional hemorrhoidectomy. Despite being an effective procedure with good long-term results, it is associated with severe pain due to the excision of the innervated anoderm below the dentate line^35^.

In 1998, a transanal circular stapling instrument, initially used for mucosal prolapses, was used to treat hemorrhoids through a procedure named stapled hemorrhoidopexy (SH)^18^ ^,^ ^25^. This technique introduced a different concept for the treatment of HD, not based on resecting the diseased hemorrhoidal cushions but on reconstituting the anatomy and physiology of the anal canal through a mucosal lift of the distal rectum^12^. Since then, several studies with follow-up of less than 5 years have reported that it is safe and efficient, with less postoperative pain, shorter hospital stay, and an earlier return to regular activities^1^ ^,^ ^22^.

This procedure has gained wide acceptance and has been performed extensively by colorectal surgeons worldwide^12^ ^,^ ^35^ ^,^ ^38^. However, results after a follow-up of more than 10 years remain unclear. Some studies have associated SH with higher rates of recurrence and the need for additional surgical procedures^33^ ^,^ ^38^. Concerns with regard to severe short-term complications including rectovaginal fistula, sepsis but also long-term complications such as anal stenosis, and fecal incontinence, have also been discussed in the literature^8^.

In the early 2000s, our group published a prospective analysis of the initial experience with SH that included 155 consecutive patients with grades III and IV symptomatic hemorrhoids operated from June 2000 to December 2003 by a single experienced colorectal surgeon, after a mean follow-up of 20 months^1^. Now, 16 years later, we reevaluated these patients and investigated the long-term complications, recurrences, and degree of satisfaction with the procedure.

## METHODS

This research was approved by the Ethics Committee of University Hospital, Universidade de São Paulo (IRB number: 44948621.5.0000.0068), and written informed consent was obtained from all patients.

In all, 98 of 155 patients who underwent SH between June 2000 and December 2003 were retrospectively reassessed, with the aim of analyzing late complications, recurrence rate, and degree of satisfaction after a long period of follow-up. In this study, we describe the results observed in the period between 2 and 16 years of follow-up, since the surgical indications, operative technical aspects, complications, and short-term results (up to 2 years) were extensively described in our previous study, published in 2006^35^. The following data were collected: sex, age, HD degree, treatments performed, clinical symptoms, complications, and degree of satisfaction with the procedure. Data collection was based on outpatient assessments or telephone interviews. The degree of satisfaction was evaluated and classified into four levels: very satisfied, satisfied, indifferent, or dissatisfied.

Statistical analysis was performed using the chi-square test for comparison of recurrence between grades III and IV hemorrhoids; p-value <0.05 was considered significant. Analysis was carried out using the SPSS version 21.0 software for Windows (SPSS, Chicago, IL, USA).

## RESULTS

From a total of 155 patients operated during the study period, 57 (36.7%) were lost to follow-up and 98 were evaluated. Demographic data and HD classification are shown in Table 1. With regard to the follow-up, 59 patients (60.2%) were interviewed by telephone and 39 (39.8%) were evaluated as outpatients.

**Table 1 -:** Patients’ demographics, degree of hemorrhoidal disease, and follow-up period.

Patient characteristics	Number
Total of patients	98 (100%)
Male/female	46 (46.9%)/52 (53.1%)
Mean age, years (range)	41 (39-79)
HD grade III	52 (53.1%)
HD grade IV	46 (46.9%)
Mean follow-up, months (range)	193 (184-231)

### Long-term results

After a very long postoperative follow-up, 16 patients (16.3%) had a recurrence of HD and all were diagnosed between 2 and 9 years following surgery. Recurrence was higher for grade IV HD (26.1%, n=12/46) than for grade III (7.7%, n=4/52) (p=0.014), but reoperation rates were similar for both groups (n=2/4 for grade III and n=9/12 for grade IV HD) (p=0.547). Of these 16 patients, 9 underwent excisional hemorrhoidectomy, 2 transanal dearterialization and mucopexy (THD-M), and the other 5 were treated with rubber band ligation in the office. All patients at the final follow-up had complete resolution of their symptoms and no further recurrences were detected.

### Long-term complications

Anal canal sub-stenosis occurred in two male patients (2.1%). Their main complaint was difficulty in eliminating stools and the need for enemas to assist on evacuation. Both patients were treated with periodic digital dilations and had complete resolution of their symptoms.

New fecal incontinence to flatus was detected in two patients (2.1%). One patient was a 47 years old female with two previous vaginal deliveries who developed new incontinence to flatus 4 years after surgery. She had excellent results with biofeedback, improving her Jorge-Wexner^15^ score from 11 to 3. The other patient was also a female with type-2 diabetes mellitus who developed new incontinence for flatus and liquid stools 10 years postoperatively and had little improvement with conservative treatment, which included dietary modification, biofeedback, and probiotics.

Additional procedures for removal of symptomatic fibrotic anal skin tags that impacted anal hygiene were required in three (3.1%) patients, all females. Chronic anal pain, rectovaginal fistula, or pelvic sepsis was not reported in this series. There was no mortality.

### Long-term satisfaction rates

Out of 98 patients who were evaluated, 34 patients (34.6%) stated that they were very satisfied, 47 (47.9%) satisfied, 7 (7.2%) indifferent, and 10 (10.3%) dissatisfied with the surgical procedure. Long-term results, late complications, and degree of satisfaction are described in Table 2.

**Table 2 -:** Long-term postoperative results, late complications, and degree of satisfaction with stapled hemorrhoidopexy.

Characteristics	N (%)
Total of patients	98 (100)
Prolapse recurrence	16 (16.3)
Skin tags	3 (3.1)
Anal sub-stenosis	2 (2.1)
Anal incontinence	2 (2.1)
Degree of satisfaction	
Very satisfied	34 (34.6)
Satisfied	47 (47.9)
Indifferent	7 (7.2)
Dissatisfied	10 (10.3)

## DISCUSSION

New surgical procedures for the surgical treatment of HD, with less invasive techniques, have been developed, such as PPH, THD-M, and LigaSure, with the aim of reducing postoperative pain and shortening the return to regular activities^36^ ^,^ ^37^.

SH has been used for more than 20 years as a therapeutic option for the third- and fourth-degree HD, mainly due to shorter operative time, less postoperative pain, and earlier return to activities when compared with conventional hemorrhoidectomy^12^ ^,^ ^35^. It is considered a safe technique especially for the treatment of circumferential hemorrhoidal prolapse, but concerns for long-term recurrences and severe complications still exists^12^ ^,^ ^20^ ^,^ ^22^ ^,^ ^29^ ^,^ ^31^ ^,^ ^35^.

Early complications, such as rectovaginal fistulas, perianal abscess, and rectal lumen obliteration, have been described extensively in the literature and are well known to colorectal surgeons^29^. However, few studies have evaluated the long-term results after more than 10 years of follow-up^4^ ^,^ ^33^ ^,^ ^38^. In our study, all 98 patients had a minimum follow-up of 184 months. There are doubts about the ideal follow-up period to assess the postoperative recurrences of non-excisional procedures (PPH and THD-M)^5^. Some authors use the following classification for the follow-up period: short, up to 2 years; medium, from 2 to 5 years; long, from 5 to 10 years; and very long, more than 10 years^38^. To the best of our knowledge, this study has the longest follow-up in the literature.

Anal sub-stenosis occurred in two patients, and both were treated successfully with periodic digital dilations. In the literature, this complication has been reported in 0.8-5% of patients after SH^9^. It is defined as circumferential narrowing of the distal rectum that cannot be transposed by a digital rectal examination. Main symptoms are difficulty in eliminating feces, the need for digital maneuvers, and the use of evacuation enemas. The diagnosis is usually straightforward with a careful digital rectal examination. The mechanism behind this phenomenon is submucosal inflammation due to ring dehiscence with local infection and full thickness excision of the rectal wall if the stapled ring is placed too deep into the anal canal with the subsequent hypertrophic scarring of the rectal wall^28^. Conservative treatment with periodic outpatient dilations and infiltration with corticosteroids is the first-line treatment. Ng et al.^23^, in a large series of 3711 cases submitted to SH, reported anal stenosis in 1.4%, most of them were successfully treated with digital dilation.

Fecal incontinence following SH is usually transient and occurs in the early postoperative period^32^. This complication is usually mild with loss of flatus or occasional soiling during exertion. It should be emphasized that fecal soiling can also occur even after conventional hemorrhoidectomy and can be triggered by diarrhea, diabetes mellitus, and medications^14^.

In our previous study^35^, three patients developed transient fecal incontinence shortly after the surgical procedure, but all of them resolved spontaneously. After prolonged follow-up, two female patients developed new fecal incontinence. This is one of the most feared complications after the treatment of HD, and it has been reported from 0 to 8% of patients following SH^24^ ^,^ ^26^ ^,^ ^32^. This complication has been attributed to internal sphincter fragmentation; a low-purse string suture that results in the staple line being too close to the pectinate line and due to mechanical anal stretching from the 33 mm stapler. Risk factors include female sex with previous vaginal delivery, pudendal neuropathy, and fecal straining^22^. The treatment is based on biofeedback rehabilitation with a success rate of up to 80%^38^. In refractory cases, the use of injectable bulking agents has been indicated^19^.

Chronic anal pain following SH is a feared complication and some authors have reported it in 1.6-17.5% patients^16^ ^,^ ^38^. In this study, no patient presented persistent pain after the long follow-up. The precise etiology is unclear, but muscle incorporation in the doughnut may play a role in its pathophysiology. Other possible causes are sphincter spasm, very low purse string including the pectinate line, rectal pocket syndrome, and chronic proctitis secondary to ischemia^7^ ^,^ ^27^.

Recurrence of prolapse with SH is highly variable and depends on the surgeon’s experience, degree of HD, and the follow-up period. Low recurrence rates have been reported in series with shorter follow-up^2^ ^,^ ^3^ ^,^ ^11^ ^,^ ^18^ ^,^ ^22^ ^,^ ^35^. A systematic review published by Shao et al.^34^ in 2008, with a follow-up ranging from 6 weeks to a median of 62 months, showed recurrence in 9% of patients, and only 7% required a new surgical procedure. White et al.^39^, in a series of 169 patients, reported that recurrences occurred in 11.2% after a mean follow-up of 15 months.

In this study, all recurrences were detected up to 9 years of follow-up, which is consistent with other series with long-term^2^ ^,^ ^3^ ^,^ ^11^ ^,^ ^13^ ^,^ ^31^ ^,^ ^34^ ^,^ ^38^ ^,^ ^39^. Our recurrence rate was 16.3%, which is lower than previously published by other authors^20^ ^,^ ^33^ ^,^ ^38^. Sturiale et al.^38^ evaluated patients after 12 years of follow-up and reported 40.9% recurrence rate. It should be noticed that evaluation was performed over the telephone and patients may have been unable to differentiate true recurrence from residual skin tags or other anorectal pathologies, which may have overestimated the recurrence rate. Schneider et al.^33^ also evaluated patients over the telephone and reported recurrence of symptoms in 47.4%. Belio et al.^4^ published the only study that performed a clinical evaluation of patients after a 10-year follow-up and reported 39% of recurrence rate.

We observed greater recurrence in HD grade IV than in grade III, similar to what was reported by other authors^13^ ^,^ ^21^. Technical aspects that may play a role in recurrence included incomplete purse string, too high a suture in the rectum, and incomplete mucosal resection in grade IV HD, which may occur when the volume of mucosal tissue exceeds the capacity of the stapler casing^6^ ^,^ ^10^ ^,^ ^14^. In the literature, recurrence after SH seems to be more frequent than after conventional hemorrhoidectomy^12^ ^,^ ^13^ ^,^ ^26^.

It is important to emphasize that although most series with long-term have recorded high recurrence rates with SH, some authors reported high recurrence with excisional methods. In a British trial with 17 years of follow-up, there was a symptomatic recurrence of HD in 26% of the patients^17^. In another large randomized study with 688 patients, the recurrence rate following Ferguson hemorrhoidectomy for grade IV HD was 40.3% (n=126/208) after a mean follow-up of 7.4 years^30^.

Besides recurrence, bothersome fibrotic skin tags are also a cause of re-intervention. In our series, three patients required removal of symptomatic skin tags after more than 24 months of follow-up. We currently recommend the excision of skin tags during the initial procedure, after the patient’s agreement. Ommer et al.^24^ in a prospective study of 224 consecutive patients concluded that the resection of large skin tags during SH provided better symptom control, lower rates of recurrence and reoperation, and higher degree of satisfaction.

Degree of satisfaction after a surgical procedure is multifactorial and subjective. Its quantification is complex and depends on the patient’s previous experiences, expectation with the surgical procedure, and the final results. A successful surgery does not always correlate with a high degree of satisfaction in terms of patient’s perspective, which has motivated the use of patients’ reported outcome in clinical trials. In our series, 82.5% were either very satisfied or satisfied with the procedure, which is consistent with other series^4^ ^,^ ^33^ ^,^ ^38^.

The main limitations of this manuscript are the retrospective nature of the study and the loss of follow-up of 36.8% of patients. However, few studies have assessed the very long-term (>10 years) results with this surgical technique. Its strengths are a large series of patients undergoing SH performed by a surgical team with experience in anorectal operations and a very long follow-up that included physical and proctologic examination in 40% of patients.

## CONCLUSIONS

This study provides evidence that SH is a safe and effective surgical procedure for the treatment of symptomatic hemorrhoids of grades III and IV, even after a long follow-up. Recurrence is higher for grade IV hemorrhoids and may occur up to 9 years of follow-up. Complications after prolonged follow-up are uncommon and most often can be managed with conservative treatment and low-complexity procedures. Reoperations for resection of new hemorrhoidal nodules are infrequent, and the patient’s degree of satisfaction with this procedure is high.
